# Annotations

**Published:** 1906-02-17

**Authors:** 


### ANNOTATIONS.

### Modern Criminology.

IN the course of an interview recently reported in the *Daily Graphic* the veteran leader of the Salvation Army told how his workers had received an almost official sanction in their work for the reclamation of convicted criminals. An enterprise so kindly and charitable cannot but command a general respect, and gains a particular interest as a concrete expression of the immense change wrought by the last few decades in the attitude of the State towards those who transgress its ordinances. There is no need to labour the intricacies of scientific criminology, for in no problem presented to sociologists is the *via media* more obscured by side-issues. It may be postulated that to reclaim the criminal and convert him into a good citizen is an action which increases the assets of the State; while on the other hand every reduction in the depth of stain left by conviction for crime is in a sense a premium placed upon lawlessness. The growing leniency of the public attitude towards criminals is in part, probably, an outcome of recent psychological advances. The idea that crime is a disease is a dramatic one, and naturally tends to invest the criminal with a halo of martyrdom in the minds of kindly and ill-informed people. And herein lies the danger, namely, that an unwise generalisation should exploit a limited truth and strike a serious blow at that belief in free-will which is the basis of government. No doubt there are people who, whether by reason of inheritance or environment, are essentially criminal from their youth up; it is regrettably certain that these are never likely to be really and permanently reclaimed; they cannot control themselves, and should be controlled by the State as being a public danger. But the commoner criminal who becomes a criminal out of a selfish disregard for his fellows can easily be over-indulged. So long as there is philanthropic work to be done among the honest but unfortunate, the reclamation of criminals will fail to appeal to a large body of thinking citizens. A cynic reading the history of Jane Cakebread, the notorious drunkard, might point a curious moral touching the rewards of vice faithfully and perseveringly served.

### The Increase of Lunacy.

SIR JOHN MCDOUGALL, giving evidence before the Royal Commission on the Care and Control of the Feeble-Minded, said he had been struck by the large increase in the number of lunatics belonging to the County of London during the last 16 years. This increase is far more than proportionate to the increase in general population. It is often said that the growing strain of modern life is responsible for the marked increase in the number of certified lunatics, and though it might be thought that this strain would not specially affect the pauper class, it must be remembered that many who in health or even in ordinary sickness are self-supporting, are forced to come upon the rates when they are permanently insane. Their families are not able to pay asylum charges, and the poor-law must step in and take the responsibility of them. Moreover, there is now a willingness which did not formerly exist to take advantage of the provision made by the Poor-law for the maintenance of paupers. Formerly insanity was looked on not merely as a misfortune to the individual, but as a disgrace to his family, and unless the patient became absolutely violent his relations put up with him. But a wiser public opinion is now arising even among the ignorant, and they are more ready to ask assistance in time. Moreover, the old idea of the asylum as a place of cruelty is dying out. Visitors have seen for themselves the provision made for the comfort, the cure, and the amusement of the inmates of asylums, and are more content to let those belonging to them go there. To this change in public sentiment we believe that much, if not all, of the apparent increase in lunacy is due. It is not that we have now so many more madmen than we had 20 years ago. It is only that fewer of them are at large.

### Uric-Acidism.

THE symptom-complex indicated by the above term (invented, so far as we know, by Dr. Robert Hutchison) is a product of the later Victorian era. It is so much a complex as to defy definition in any less compass than a monograph; but definition is quite unnecessary, for all educated persons, except those of the faculty, are experts in the disorder. Those of us who wish to learn something definite about this mysterious malady need only keep their ears open at any place of entertainment, at any club, or any dinner-party, in order to gain dogmatic instruction upon the hydra-headed monster, its disguises and subtleties, and the defences essential to its repulse. We have mentioned the disguises of uric-acidism, for the custom of it is to appear in disguise. By comparison with the total frequency of its appearances, the number of occasions upon which it may be seen unmasked—in the form, that is to say, of urinary gravel—is almost insignificant. Far more often we find it expressing its presence by means of headache, or backache, or "liver," or, in short, by any of the thousand and one subjective anomalies whose organic counterpart has so far escaped the morbid anatomist and pathological chemist; and the lay cures for all its varieties are dietetic modifications. We have no title, as a profession, to complain of this circumstance, for so long as mysteries exist humanity will grope for truth, and will accept as a prophet every propagandist who can materialise his creed in the matter at issue so as to be understanded of the people. We must sadly confess that until a very recent period dietetics have suffered an undeserved neglect at the hands of science. This is a reproach, for the profession should lead in such things instead of being driven by lay competition. No doubt most of us feel that man, the omnivore, the most adaptable feeder in the creation, must be above such trifling disabilities as are implied by uric-acidism; but man, the natural animal, is a very different creature from man the slave of stool and office. Specialisation often means loss of vigour, and it is quite possible that idiosyncrasy towards foodstuffs is exaggerated by sedentary days.

